# Low Dietary Fish Meal Induced Endoplasmic Reticulum Stress and Impaired Phospholipids Metabolism in Juvenile Pacific White Shrimp, *Litopenaeus vannamei*

**DOI:** 10.3389/fphys.2020.01024

**Published:** 2020-08-18

**Authors:** Shiwei Xie, Yongjian Liu, Lixia Tian, Jin Niu, Beiping Tan

**Affiliations:** ^1^Laboratory of Aquatic Animal Nutrition and Feed, Fisheries College, Guangdong Ocean University, Zhanjiang, China; ^2^School of Life Sciences, Institute of Aquatic Economic Animals, Sun Yat-sen University, Guangzhou, China

**Keywords:** endoplasmic reticulum stress, immune response, lipid metabolism, metabolomics, sterol

## Abstract

This study mainly evaluated the low dietary fish-meal (FM) on growth performance, immune competence and metabolomics response of juvenile Pacific white shrimp, *Litopenaeus vannamei* reared at low salinity (7‰). Five experimental diets with graded levels (25, 20, 15, 10, and 5%) of FM were formulated. Weight gain, feed utilization and survival were decreased with the decreasing FM levels. When dietary FM decreased, glucose, cholesterol, total bile acids, and triglyceride in hemolymph decreased. Fatty acid synthesis was promoted and fatty acid lipolysis was reduced in hepatopancreas of shrimp fed low dietary FM. Endoplasmic reticulum (ER) stress related genes expression in hepatopancreas were down-regulated and in intestine were upregulated by low dietary FM. *Inhibitor kappa B kinase*β expression in intestine increased with the dietary FM levels, while mRNA levels of *dorsal* in hepatopancreas showed the opposite tendency. Hematoxylin and eosin (H&E) stain and transmission electron microscope analysis of intestinal samples indicated that low FM diets induced intestinal morphological damage, ER swollen and chromatin condensation. UPLC-Q/TOF-MS analysis indicated that degree of unsaturation of the fatty acid chains of phospholipids in hemolymph decreased with the decreasing dietary FM levels. Lysophospholipids and bile acids metabolism were disturbed by high levels of FM sparing in diet. These results indicated when dietary FM contents decreased, ER stress of shrimp was induced. The decreased unsaturated degree of phospholipids, decreased contents of lysophospholipids, altered lipid metabolism and ER stress may responsible for the impaired growth performance and health of shrimp fed a low FM diet.

## Introduction

Fish meal (FM) is a critical ingredient in aquatic feeds due to its balanced nutritional composition (Bendiksen et al., 2011; Olsen and Hasan, 2012; Xie et al., 2014). While the supply of FM was short recently, which make FM to be a limiting factor for the sustainable development of aquaculture. *Litopenaeus vannamei* has been one of the biggest consumers of FM (Tacon and Metian, 2008; Olsen and Hasan, 2012), the past 20 years witnessed plentiful research about FM replacement in *L. vannamei*, which showed that FM could be partial substituted without deleterious effects (Amaya et al., 2007; Ye et al., 2011; Yue et al., 2012; Kuhn et al., 2016). Excessive substitution of FM generally led to poor growth performance, which may because the nutrient-sensing and metabolism of fish and shrimp are changed when fed a low FM diet (Wang et al., 2016; Xu et al., 2016). Besides growth retardation, shrimp health were impaired when fed a low FM diet (Wu et al., 2009; Xie et al., 2016, 2020), figure out the potential mechanism would be helpful in further reducing FM in aquafeeds, not only in the feed of *L. vannamei*, but also in the feeds of many carnivorous fish. The wide nutritional differences between FM and other ingredients make it hard to find the key metabolism pathways and biomarkers which responsible for the negative effects brought by low dietary FM.

Metabolomics is to analyze a complete set of low molecule metabolites in biological samples such as plasma, urine and cell (Johnson et al., 2016). Because metabolites are hypersensitive to exogenous factors such as diet, metabolomics has become a strong discovery tool in nutrition research (Putri et al., 2013). Usually, metabolomics studies of biofluids and tissues are widely performed to evaluate the influences of dietary composition on metabolism and health of human and animals (He et al., 2008). In aquaculture nutrition research, metabolomics had been applied to evaluate nutritional requirements and ingredients utilization, and tend to be a strong technology to detect the complex nutrient utilization differences and identify the metabolic biomarkers (Jin et al., 2015; Li et al., 2017b; Ma et al., 2017; Wei et al., 2017; Alfaro and Young, 2018; Xie et al., 2018).

The commonly used metabolomics platforms are gas chromatography couple with mass spectrometry (GC-MS), liquid chromatography couple with mass spectrometry (LC-MS), and NMR spectroscopy (Rezzi et al., 2013). Recently, applying high pressure resistant column, ultra-performance liquid chromatography coupled with quadrupole time of flight mass spectrometry (UPLC-Q/TOF-MS) has demonstrated significant advantages in measurement time, resolution, throughput, sensitivity and specificity, and shown to be a powerful analytical technology for metabolomics research (Qian et al., 2017; Rindlisbacher et al., 2018).

Like other animals, crustaceans have different lipid classes which including fatty acids, triacylglycerides, phospholipids and sterols (Sinha et al., 2018). Lipids are not only serve as the major energy source and the carrier of fat-soluble vitamins, but also act as the components of cell membranes and the precursors of many important metabolites, therefore lipids are critical to the growth, reproduction, osmoregulation, membrane fluidity, and immune response of animals (González-Félix et al., 2002; Arts and Kohler, 2009; Xu et al., 2018).

In this study, metabolomics concept was employed to evaluate the relationship between growth performance, lipid metabolism, immune-related genes and histoarchitecture of gut of *L. vannamei* fed different dietary FM levels.

## Materials and Methods

### Diet Preparation

Five experimental diets were formulated with graded levels of FM (25, 20, 15, 10, and 5%), FM was replaced by chicken meal, wheat gluten, soy protein concentrate and blood meal. Diet formulation and proximate composition are given in Table 1, amino acid and fatty acid composition of the diets are given in Supplementary Table 1 and Supplementary Table 2, respectively. Crystalline amino acids were precoated with carboxymethyl cellulose to prevent leaching loss (Xie et al., 2014). The pellets (1.2 mm diameter) were cold-extruded using a pelletizer (South China University of Technology, Guangdong, China), then heated for 60 min in an electric oven (70°C), and air-dried to approximately 10% moisture, finally stored at -20°C before used (Xie et al., 2018).

**TABLE 1:** Formulation and proximate of experimental feeds (% dry matter).

Ingredients	1	2	3	4	5
Fish meal^a^	25	20	15	10	5
Soybean meal^a^	25	25	25	25	25
Peanut meal^a^	10	10	10	10	10
Wheat flour	23.30	23.30	23.30	23.30	23.30
Beer yeast^a^	4	4	4	4	4
Shrimp meal	2	2	2	2	2
Chicken meal^a^	2	2.25	2.5	2.75	3
Blood meal^a^	0	0.85	1.7	2.55	3.4
Soy protein concentrate	0	1.06	2.11	3.17	4.22
wheat gluten	0	1.2	2.4	3.6	4.8
Fish oil^a^	1.2	1.65	2.1	2.55	3
Soy oil^a^	1.2	1.2	1.2	1.2	1.2
Soy lecithin	1	1	1	1	1
Vitamin mixture^c^	1	1	1	1	1
Mineral mixture^c^	1	1	1	1	1
Monocalcium phosphate^a^	1	1.3	1.6	1.9	2.2
Choline chloride (50%)^c^	0.2	0.22	0.24	0.26	0.28
Vc phosphate^c^	0.1	0.1	0.1	0.1	0.1
Carboxyl Methyl Cellulose	2	2	2	2	2
BIO-MOS^d^	0	0.1	0.2	0.3	0.4
Phytase^e^	0	0.01	0.02	0.03	0.04
Met-Met^f^	0	0.07	0.14	0.21	0.28
L-lysine monohydrochloride^g^	0	0.13	0.26	0.39	0.52
Threonine^g^	0	0.06	0.12	0.18	0.24
Arginine^g^	0	0.07	0.14	0.21	0.28
Glycine^g^	0	0.1	0.2	0.3	0.4
Alanine^g^	0	0.08	0.16	0.24	0.32
Histine^g^	0	0.01	0.02	0.03	0.04
Valine^g^	0	0.05	0.1	0.15	0.2
GABA^g^	0	0.005	0.01	0.015	0.02
Taurine^g^	0	0.05	0.1	0.15	0.2
Leucine^g^	0	0.06	0.12	0.18	0.24
Isoleucine^g^	0	0.07	0.14	0.21	0.28
Ornithine^g^	0	0.004	0.008	0.012	0.016
Vitamin B12^c^	0	0.000002	0.000004	0.000006	0.000008
Nicotinic acid^c^	0	0.0005	0.001	0.0015	0.002
Vitamin K^c^	0	0.00002	0.00004	0.00006	0.00008
ZnSO_4_.7H_2_O^c^	0	0.0025	0.005	0.0075	0.01
FeSO_4_.7H_2_0^c^	0	0.003	0.006	0.009	0.012
KI^c^	0	0.00003	0.00006	0.00009	0.00012
Na_2_SeO_3_^c^	0	0.000015	0.00003	0.000045	0.00006
**Proximate composition**					
Dry matter	89.30	89.75	89.29	89.21	89.05
Crude protein	42.32	42.11	41.97	42.15	42.08
Crude lipid	7.45	7.64	7.39	7.52	7.48
Ash	9.84	9.46	9.66	9.53	9.59

*^*a*^Supplied by Xingxing Feed Corporation, Guangzhou, China. ^*b*^Supplied by Wilmar, Beijing, China. ^*c*^Supplied by Ashare, Guangzhou, China. Compositions (kg^–^*^1^ of mixture)*: riboflavin, 750 mg; pyridoxine HCL, 500 mg; vitamin A, 250,000 IU; thiamin, 500 mg; cyanocobalamin, 1 mg; menadione, 250 mg; folic acid, 125 mg; myo-inositol, 2,500 mg; biotin, 10 mg; a-tocopherol, 3,750 mg; calcium pantothenate, 1,250 mg; nicotinic acid, 2,000 mg; vitamin D3, 45,000 IU; vitamin C, 7,000 mg, Zn, 4,000 mg; K, 22,500 mg; I, 200 mg; Co, 50 mg; NaCl, 2.6 g; Mg, 3,000 mg; Cu, 500 mg; FeSO_4_, 200 mg; Se, 10 mg. ^*d*^Supplied by Alltech, Beijing, China, derived from a selected strain of Saccharomyces cerevisiae yeast. ^*e*^Supplied by Yiduoli Biotechnology co., Ltd., Zhuhai, China, produced from fungus, 2000 IU g^–^*^1^.*^*f*^Supplied by Evonik, Beijing, China. ^*g*^Supplied by Aladdin Industrial Corporation, Shanghai, China.*

### Shrimp and Experimental Conditions

Juvenile *L. vannamei* were purchased from a commercial hatchery (Xiangzhou district, Zhuhai, China). Shrimp were acclimated for 3 weeks and fed a commercial shrimp diet (43.67% crude protein, 8.17% crude lipid, Haida Group, Guangdong, China). Prior to the experiment, 600 shrimp (0.3 g) were randomly assigned into 20 tanks (cylindrical fiberglass, 300 L), and fed one of the five diets in quadruplicate for 8 weeks. Shrimp were fed three times per day at 8:00, 13:00, and 18:00 to apparent satiation. Uneaten feed was collected an hour after the feeding by siphoning in order to determine feed efficiency.

Throughout the feeding trial, temperatures of water was 27.23 ± 1.41°C, the salinity was 7.02 ± 0.21‰, pH was 7.81 ± 0.07, the ammonia nitrogen was 0.03 ± 0.01 mg L^–1^, and dissolved oxygen was 6.88 ± 0.25 mg L^–1^. The parameters of water quality were collected twice weekly using the commercial kits (Sangpu, Beijing, China). The photoperiod was natural during the husbandry trial (28th, April-22th, June).

### Sampling and Chemical Assays

At the end of feeding phase, shrimp were overnight fasted, shrimp from each tank were weighed and counted. Hemolymph from 10 shrimp per tank were taken from the pericardial cavity using a 1-ml sterile syringe (no anti-coagulant was used), pooled and mashed, then centrifuged (8000 rpm, 4°C, 10 min). The supernatant was collected and stored at -80°C. Intestine and hepatopancreas from two shrimp per tank were immediately frozen and stored at -80°C for RNA extraction.

Moisture, crude lipid, crude protein, and ash contents in the diets were determined using standard methods (AOAC, 1995). Fatty acid compositions of diets were determined by China National Analytical Center. Lipids were extracted by a mixture of chloroform and methanol (2:1, v/v), then solvents was evaporated and fatty acids was saponificated by potassium hydrate. Fatty acids methyl esters were separated and quanitified by a gas chromatograph (GC 7820A, Agilent, United States) equipmented with a detector flame ionization (FID) and a HP-88 column (long × inner diameter × film thickness: 30 m × 0.25 mm × 0.2 μm) with hydrogen as carrier gas. The temperature gradient was from 100°C to 180°C at 10°C/min and then to 200°C at 1°C/min and finally to 230°C at 4°C/min, each sample was running for 49.5 min.

### Hemolymph Biochemical Index Assays

Concentrations of glucose (GLU), triglyceride (TG), total bile acid (TBA), and cholesterol (CHO) in hemolymph were determined by glucose oxidase-peroxidase method (Sharp, 1972), glycerol-3-phosphate oxidase-peroxidasemethod (Kohlmeier, 1986), 3α-hydroxysteroid dehydrogenase method (Bruusgaard et al., 1977) and cholesterol oxidase-peroxidase method (Allain et al., 1974), respectively. These indexes were measured following the instructions of the detection kits (Nanjing Jiancheng Bioengineering Institute, China), through monitoring the absorbance changes at 505, 510, 405, and 510 nm in a microplate reader (TECAN infinite M200, Switzerland), respectively.

### Intestinal Histology Analysis

In each tank, the similar part of midgut from three shrimp were firstly fixed in Bouin’s solution (within 24 h), secondly transfered in 70% ethanol. Then samples were dehydrated in a graded series of ethyl alcohol and embedded in paraffin. Sections were stained with hematoxylin/eosin and observed under a microscope (Nikon Ni-U, Japan) at the magnification of 100×.

Another three midgut per tank were collected for transmission eletron microscopy (TEM) examination. The method was described by Zhang et al. (2012) and Xie et al. (2020), briefly, samples were fixed in 2.5% glutaraldehyde solution (4°C), then fixed in 1% osmium tetroxide (OsO4) for 1 h, dehydrated in a graded series of ethyl alcohol, finally embedded with resin. Ultrathin sections (90 nm) were placed on copper grids and were stained with saturated uranyl acetate solution for 30 min, rinsed with distilled water and poststained with lead citrate for 30 min. Ultrathin sections were screened and observed with a TEM (FEI Tecnai G2 20, Holland).

### RNA Extraction and qRT PCR Analysis

The methods of RNA extraction, cDNA synthesis and qRT PCR were described as Xie et al. (2019). Total RNA was extracted from hepatopancreas and intestine using Trizol^®^ reagent (Invitrogen, United States). Agarose gel electrophoresis was used to assess RNA integrity, and concentration and purity of RNA was measured by spectrophotometric analysis (A260:A280 nm). cDNA was synthesized using a PrimeScriptTM RT reagent kit with gDNA Eraser (Takara, Japan).

Real-time PCR for the genes were measured on the LightCycler 480 (Roche Applied Science, Basel Switzerland) using SYBR^®^ Premix Ex Taq^TM^ II (Takara, Japan). The efficiency of the primers were close to 100% (Supplementary Table 3).

Normalization of qRT PCR data was done by geometric averaging of tworeference genes (β-actin and elongation factor1α) (Vandesompele et al., 2002). Each sample were runned three technical replications.

### UPLC-Q/TOF-MS Analysis

Thirty six shrimp hemolymph samples for all treatments were analyzed by UPLC-Q/TOF-MS in this research to study the lipid profiles in FM25, FM15 and FM5, 12 for each treatment. The method was described by Xie et al. (2020). Briefly, 400 μL acetonitrile/methanol (9:1) was mixed with 100 μL hemolymph and vortexed for 30 s. The mixture was centrifuged at 13, 000 g and 4°C for 10 min, 200 μL supernatant was used for UPLC-Q/TOF-MS analysis. Equal amounts of all samples were pooled as a QC sample for UPLC-Q/TOF-MS system conditioning and quality control.

UPLC-Q/TOF-MS was performed on an ACQUITY UPLC system (Waters, Manchester, United Kingdom) coupled with a G2-Si HDMS QTOF mass spectrometer (Waters, Manchester, United Kingdom). Chromatographic separation was carried out at 40°C on an ACQUITY HSS T3 1-class column (2.1 × 100 mm, 1.8 μm, Waters). The method of the UPLC-Q/TOF-MS analysis was same as Xie et al. (2020).

Peak detection and alignment of the mass dada were performed by Waters Masslynx v4.1, then all data were normalized to the summed total ion intensity per chromatogram. Four thousand twenty-five and three thousand two hundred thirty-three features were detected in positive and negative modes, respectively. All the features with CVs >30% were removed. Principal component analysis (PCA) and orthogonal partial least squares discriminant analysis (OPLS-DA) were performed by SIMCA-P (Version 13.0, Umetrics, Sweden).

The significantly different metabolites were selected based on variable influence on projection (VIP) values obtained from the OPLS-DA model, *P*-values from one way ANOVA analysis and the maximum fold change. The metabolites with VIP values larger than 1.0, *P*-values less than 0.05 and fold change larger than 1.5 were regarded as significantly different metabolites.

Metabolite indentification was performed using Progenesis QI (Waters, Non-linear Dynamics, Newcastle, United Kingdom). The Human Metabolome databased (HMDB), LipidMaps and Chemspider were used for MS1 identification, and theoretical fragments were used for MS/MS identification. The score (total 60) of identified metabolites obtained from QI larger than 40 was regarded as acceptable, otherwise would be regarded as uncertain.

Pheatmap package based on R software (version 3.7.2) was used for heatmap and cluster analysis.

### Calculations and Statistical Analysis

The parameters were calculated as follows:

Percent weight gain (WG,%) = 100 × (final body weight - initial body weight)/initial body weight

Survival (%) = 100 × (final number of shrimp)/(initial number of shrimp)

Specific growth ratio (SGR,% day^–1^) = 100 × (Ln final body weight - Ln initial body weight)/t

Feed conversion ration (FCR) = feed consumed (g, dry weight)/weight gain (g, wet weight)

Protein efficiency ratio (PER) = weight gain (g, wet weight)/protein intake (g, dry weight)

Protein productive value (PPV,%) = 100 × protein gain (g)/protein intake (g)

All the data were presented as the means with SEM and were statistically analyzed by SPSS 19.0 (SPSS, Chicago, IL, United States). Data were first tested for normality (Shapiro-Wilk test) and homogeneity (Levene’s test). Normal distribution data were analyzed by one-way ANOVA. When data showed significant differences (*P* < 0.05), they were further compared using Duncan’s multiple range test., Non-parameter Kruskal-Wallis test was applied when data did not have a homogeneous variance, followed by all pairwise multiple comparisons if there were significant differences (*P* < 0.05).

## Results

### Effect of Dietary FM Levels on Growth Performance of White Shrimp

**FIGURE 1:**
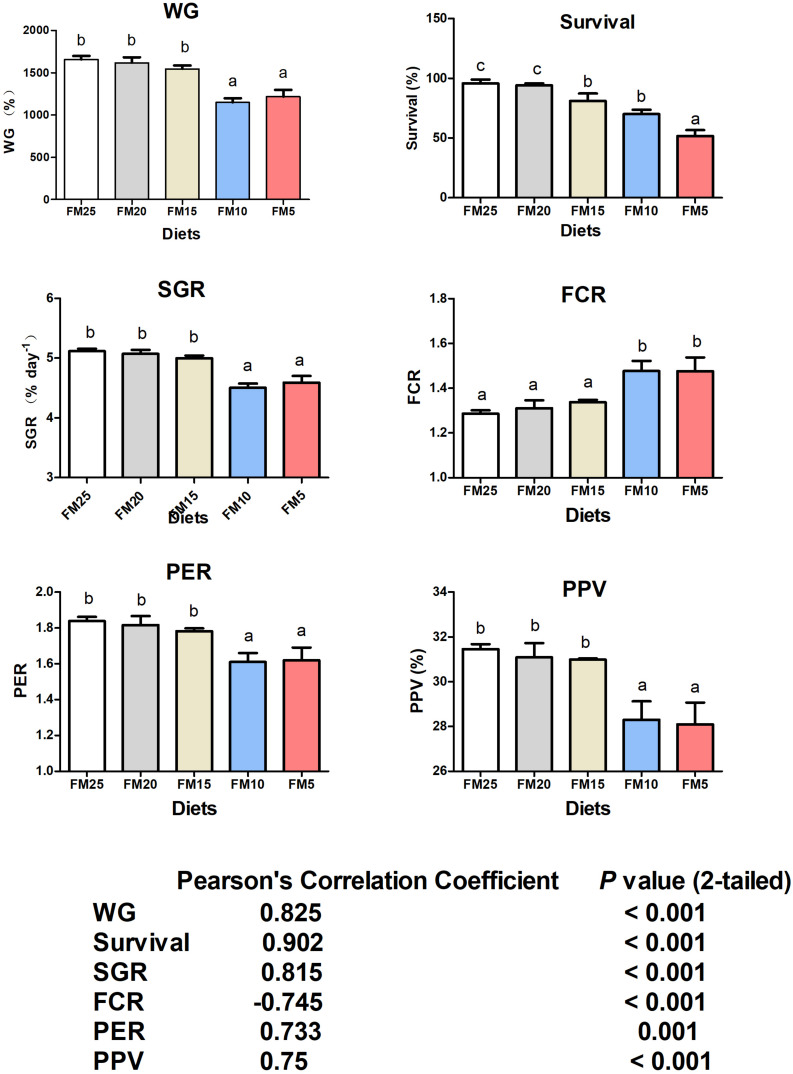
The effect of fish meal levels on growth performance and feed utilization of white shrimp. Data are means with SEM. Mean values with different letters denote significantly differences (*P* < 0.05). WG, weight gain; SGR, specific growth ratio; PER, protein efficiency ratio; FCR, feed conversion ration; PPV, protein productive value.

WG, survival, SGR, FCR, PER, and PPV were significantly affected by dietary FM levels (*P* < 0.05) (Figure 1). WG, SGR, FCR, PER, and PPV were lower in shrimp fed diets contained 10 and 5% FM, survival was significantly decreased when dietary FM levels were less than 20% (Figure 1). After dietary FM decreased from 25 to 5%, FCR increased 14.79%, WG, SGR, PER, PPV and survival decreased 26.55, 10.25, 11.94, 10.67, and 46.09%, respectively. All the parameters were significantly correlated to FM levels in the diet.

### Glucose and Lipid Metabolism Were Affected by FM Replacement

**FIGURE 2:**
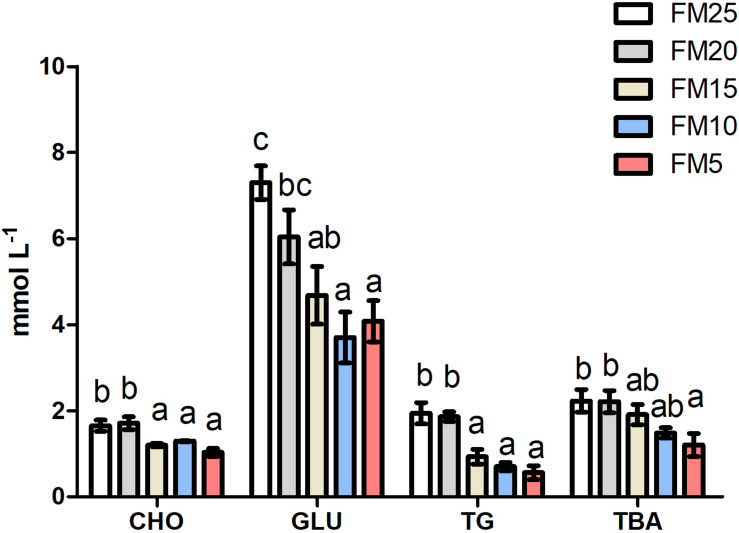
The effect of fish meal levels on hemolymph parameters of white shrimp. GLU, glucose; TG, triglyceride; CHO, cholesterol; TBA, total bile acid. Data are means with SEM. Mean values with different letters denote significantly differences (*P* < 0.05).

Effect of FM replacement on hemolymph biochemical indexes of *L. vannamei* was displayed in Figure 2. Glucose was significantly lower in shrimp fed diets with FM levels less than 20% compared to those fed FM25 and FM20 (*P* < 0.05). Triglyceride and cholesterol showed similar tendency with glucose, higher in shrimp fed FM25 and FM20 compared to shrimp fed other diets (*P* < 0.05). Total bile acid (TBA) was higher in shrimp fed FM25 and FM20 than those fed FM5 (*P* < 0.05).

**FIGURE 3:**
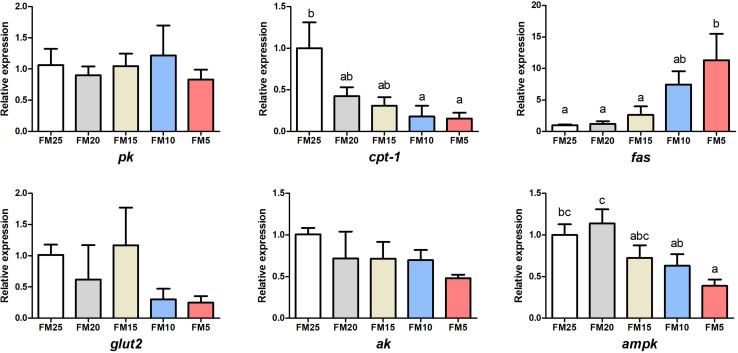
The effect of fish meal levels on energy metabolism of white shrimp. Pk, pyruvate kinase; ak, arginine kinase; cpt-1, carnitine palmitoyltransferase 1; fas, fatty acid synthetase; ampk, denosine monophosphate activated. Data are means with SEM. Mean values with different letters denote significantly differences (*P* < 0.05).

The gene expression of *fatty acid synthetase* (*fas*) was increased with the decreasing dietary FM levels (*P* < 0.05) (Figure 3), while the expression of *carnitine palmitoyltransferase 1* (*cpt-1*) and *denosine monophosphate activated protein kinase* (*ampk*) showed opposite tendency, which were higher in shrimp fed high dietary FM than shrimp fed other diets (*P* < 0.05). There were no differences in the mRNA levels of *pyrubate kinase* (*pk*), *glucose transporter 2* (*glut2*) and *arginine kinase* (*ak*) among the five groups (*P* > 0.05).

### Low FM Diet Induced Endoplasmic Reticulum Stress (ERS) of Shrimp

**FIGURE 4:**
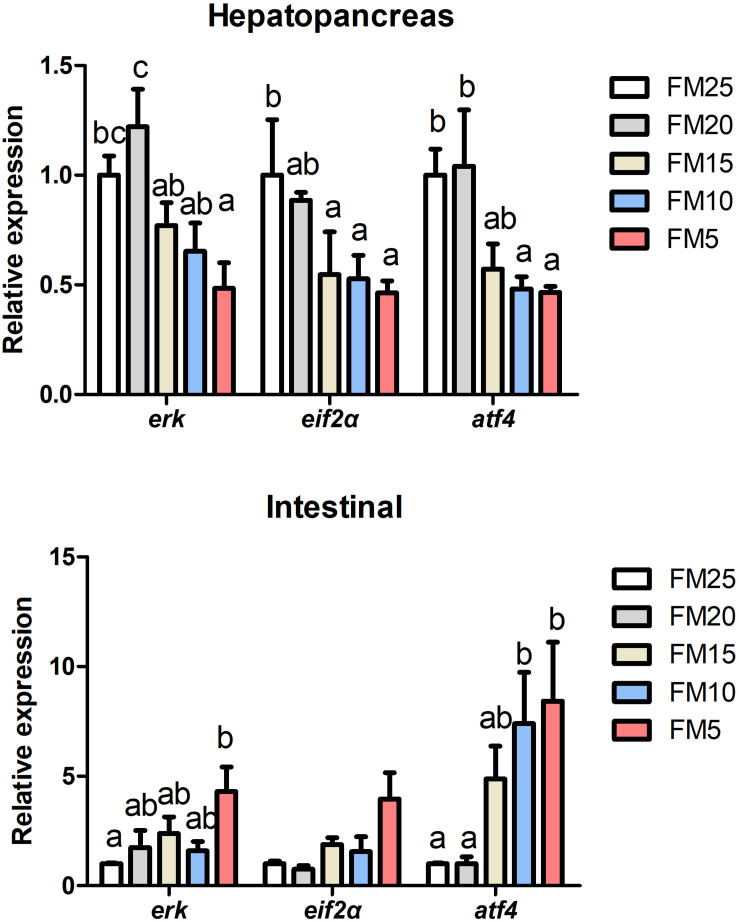
The effect of fish meal levels on mRNA expression of ER stress related genes of white shrimp. eif2α, eukaryotic initiation factor 2 alpha; atf4, activating transcription factor 4; erk, extracellular signal-regulated kinase. Data are means with SEM. Mean values with different letters denote significantly differences (*P* < 0.05).

*Activating transcription factor 4* (*atf4*), *extracellular signal-regulated kinase* (*erk*) and *eukaryotic initiation factor 2 alpha* (*eif2*α) mRNA levels in hepatopancreas were decreased with the decreasing dietary FM levels (Figure 4) (*P* < 0.05), while the expression of these three genes in intestine showed the opposite tendency as the hepatopancreas. *Erk* expression was lower in shrimp fed FM25 compared to those fed FM5, and *atf4* mRNA levels were lower in shrimp fed FM25 and FM20 compared to those fed diet contains lowest FM (*P* < 0.05).

### Low FM Diets Impaired Immune Ability of Shrimp and Induced Intestinal Damage

**FIGURE 5:**
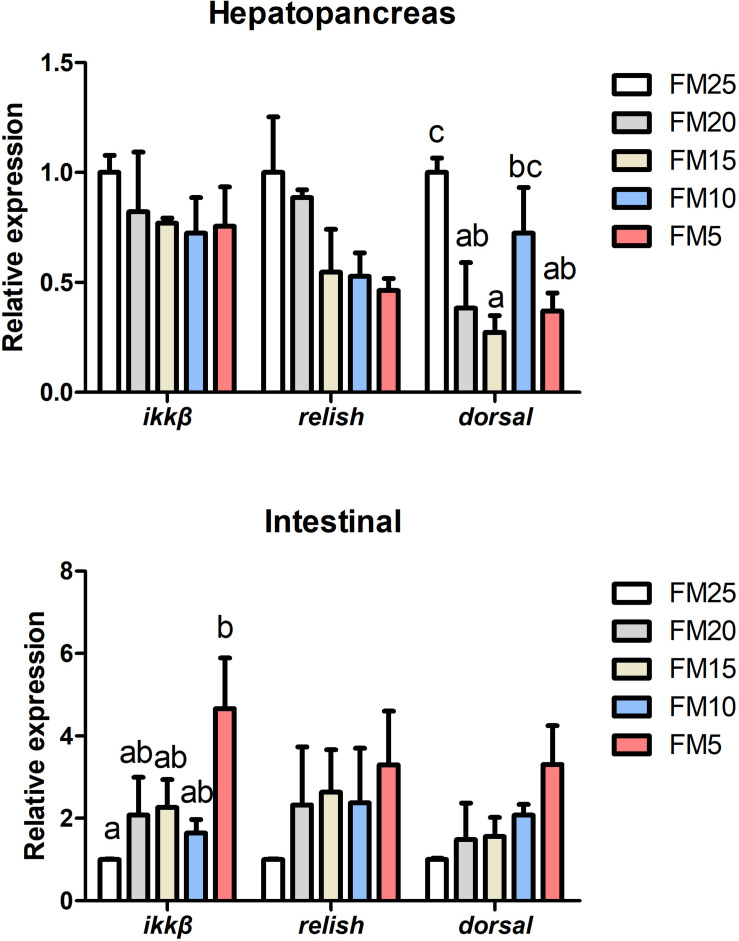
The effect of fish meal levels on mRNA expression of NF-kB pathway related genes of white shrimp. Ikkβ, inhibitor of nuclear factor kappa-B kinase β. Data are means with SEM. Mean values with different letters denote significantly differences (*P* < 0.05).

*Dorsal* mRNA levels in hepatopancreas were significantly affected by FM levels in the diets, the highest *dorsal* expression was in shrimp fed FM25 and lowest was in shrimp fed FM15 (*P* < 0.05) (Figure 5), there were no differences in the expression of *ikk*β and *relish* among all the treatments (*P* > 0.05). *Ikk*β mRNA expression in intestine was increased with the decreasing dietary FM levels (*P* < 0.05), there were no differences in the expression of *dorsal* and *relish* among the five groups (*P* > 0.05).

**FIGURE 6:**
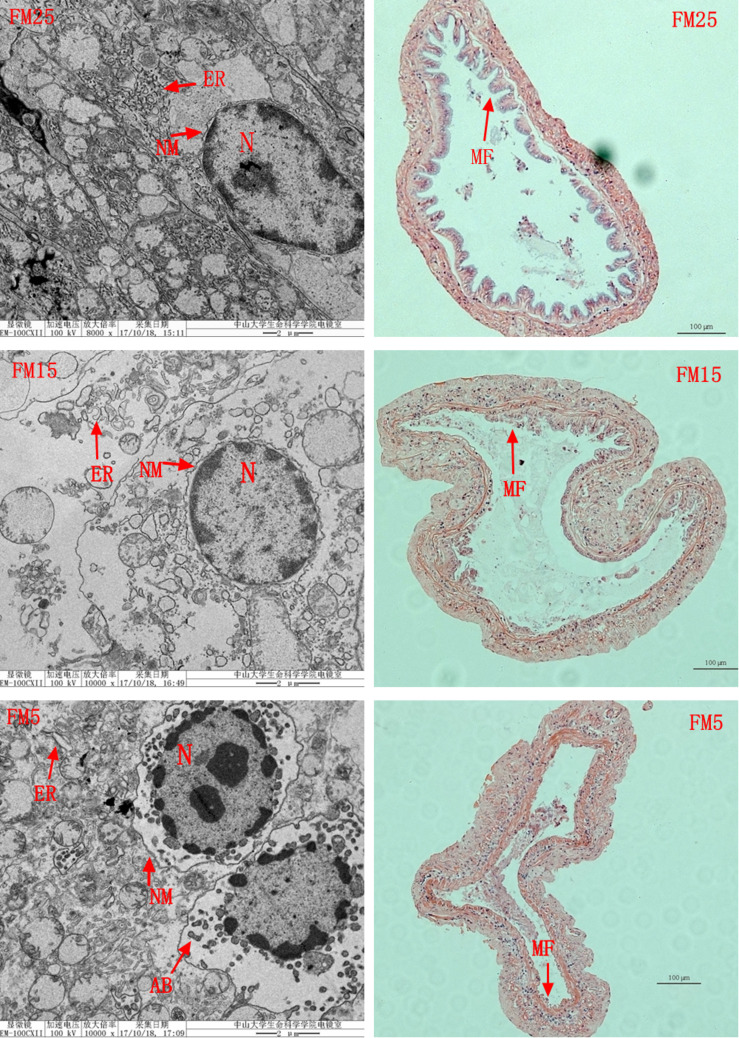
The effect of fish meal levels on intestinal morphology of white shrimp. ER, endoplasmic reticulum; N, cell nucleus; NM, nucleus membrane; AB, apoptosis bodies; MF, mucosal folds. Intestinal HE stain (the right part) showed that the intestinal MF were clearly impaired in shrimp fed the low FM diets (FM15 and FM5). TEM analysis (the left part) showed the ER was swollen in shrimp fed the FM15 or FM5. The outside NM was partly separated from the inside NM in shrimp fed the FM15, and they were totally separated in shrimp fed the FM5. The apoptosis bodies were formed in shrimp fed the FM5 diet.

Intestinal HE stain showed that intestinal mucosal folds were clearly impaired in shrimp fed the low FM diets (FM15 and FM5) (Figure 6). TEM results showed that the intestine suffered more serious damage in shrimp fed the low FM diets (Figure 6), ER was swollen in shrimp fed the FM15 and FM5. The outside nuclear membrane was partly separated from the inside nuclear membrane in shrimp fed FM15 diet, and they were totally separated in shrimp fed FM5 diet. The apoptosis bodies were formed in shrimp fed the FM5 diet.

### Multivariate Analysis and Identification of Significant Different Metabolites

**FIGURE 7:**
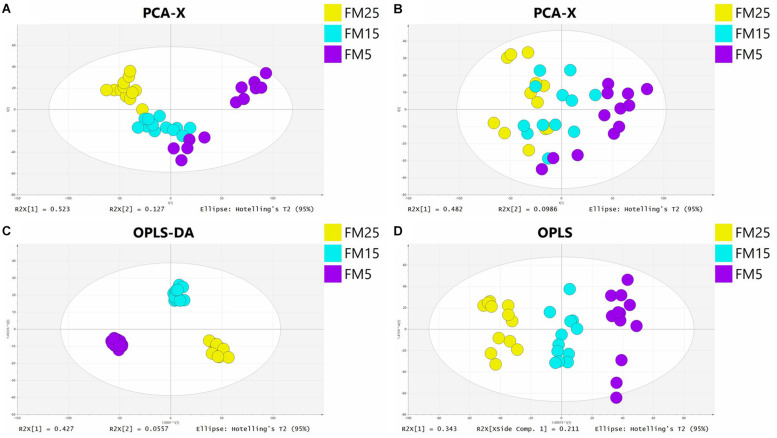
The principal component analysis (PCA) score plot in ESI+ **(A)** and ESI− **(B)** models and orthogonal partial least squares discriminant analysis (OPLS-DA) score plot in ESI+ **(C)** and ESI− **(D)** models.

In this study, PCA analysis showed a clear cluster in ESI+ (*R_2_X* = 0.808, *Q_2_* = 0.708) and ESI- (*R_2_X* = 0.84, *Q_2_* = 0.668) modes (Figures 7A,B). OPLS-DA analysis clearly distinguished the different groups in ESI+ (*R_2_X* = 0.816, *R_2_Y* = 0.979, *Q_2_* = 0.884) and ESI- (*R_2_X* = 0.627, *R_2_Y* = 0.969, *Q_2_* = 0.927) modes (Figures 7C,D), showed a goodness of fit (*R*_2_) and predictive ability (*Q*_2_) in this model. Features with VIP values larger than 1.0, *P*-values less than 0.05 and fold change larger than 1.5 were regarded as significantly different features. Altogether, there were 545 significant features satisfying the criterion (309 in ESI+ and 236 in ESI-), 74 in ESI+ and 137 in ESI- were identified, 26 in ESI+ and 67 in ESI- were regarded as credible. In total, 93 significant different metabolites were identified in this study (Table 2).

**TABLE 2:** The differential metabolites identified in hemolymph.

Metabolites	m/z	RT(min)	Adducts	Formula	Score	*P*	FM15/FM25	FM5/FM25
**Positive**								
PC(18:0/0:0)	524.3703	11.55	M + H	C26H54NO7P	48.7	0.00	0.45	0.29
PC(14:0/20:5)	752.5216	14.33	M + H	C42H74NO8P	53.9	0.00	0.48	0.15
PC(18:2/20:5)	826.5355	14.57	M + H	C46H78NO8P	44.4	0.00	0.47	0.17
PC(14:1/22:6)	776.5221	14.7	M + H	C44H74NO8P	48.3	0.00	0.63	0.28
PC(P-16:0/18:4)	760.5224	14.71	M + H	C42H76NO7P	52.6	0.00	0.44	0.18
PC(14:0/18:2)	820.5112	14.76	M + H	C40H76NO8P	45	0.00	0.77	0.43
PC(16:1/18:2)	778.5346	14.87	M + H	C42H78NO8P	52.4	0.00	0.78	0.40
PE(18:3/22:4)	790.5397	14.95	M + H	C45H76NO8P	42.1	0.00	0.76	0.40
PC(16:0/22:6)	828.5493	15.19	M + H	C46H80NO8P	50.8	0.00	0.57	0.28
PC(14:0/18:1)	754.5335	15.27	M + H	C40H78NO8P	42.6	0.00	0.88	0.56
PC(18:2/22:5)	854.5643	15.28	M + H	C48H82NO8P	48.8	0.00	0.53	0.25
SM(d17:1/17:0)	725.5538	15.31	M + H	C39H79N2O6P	53.5	0.00	1.43	1.60
PC(P-16:0/20:4)	788.5516	15.37	M + H	C44H80NO7P	54.6	0.00	0.59	0.28
PS(18:0/20:3)	814.5637	15.69	M + H	C44H80NO10P	48.1	0.00	0.71	0.43
PE(18:0/22:6)	840.5783	15.75	M + CH3OH + H	C45H78NO9P	51	0.00	0.53	0.31
LacCer(d18:1/14:0)	816.5819	15.93	M + H - H2O	C44H83NO13	40.4	0.00	0.60	0.30
LacCer(d18:1/14:0)	856.5773	15.98	M + H	C44H83NO13	41.5	0.00	0.59	0.35
PC(20:3/P-18:1)	794.5981	16.25	M + H	C46H84NO7P	45.2	0.00	0.58	0.38
PS(21:0/20:5)	884.5988	16.59	M + CH3OH + H	C47H82NO10P	46.1	0.00	0.60	0.39
LacCer(d18:1/16:0)	844.6112	16.97	M + H - H2O	C46H87NO13	44.1	0.00	0.46	0.21
Oceanalin A	864.6049	18.01	M + H	C41H72N2O9	52.2	0.00	1.59	2.18
DAT(16:0/23:0)	927.7087	18.15	M + H - H2O	C53H100O13	44.2	0.00	12.09	12.89
Cer(d18:1/24:1)	912.6259	18.18	M + H - H2O	C48H91NO11S	42.7	0.00	0.75	0.55
PC(22:0/16:1)	838.6227	18.34	M + H	C46H90NO8P	44	0.00	1.65	1.82
TG(18:4/18:4/20:4)	927.7082	18.67	M + CH3OH + H	C59H90O6	40.1	0.00	5.25	4.77
**Negative**								
Diethylenetriamine	509.2746	1.99	2M - H	C14H22ClNO	48.9	0.00	0.89	3.04
Crustecdysone	525.3059	2.12	M - H, M + FA - H	C27H44O7	57.6	0.00	0.89	3.87
2-ethyl butanoate	258.0793	2.21	M + FA - H	C10H15NO2S	51.8	0.00	0.49	0.11
Monocrotaline	324.1474	2.21	M - H	C16H23NO6	48.7	0.01	1.54	1.45
Macaridine	260.0941	2.31	M + FA - H	C13H13NO2	54	0.00	0.43	0.16
5-Amino-benzopyran-4-one	306.1371	2.38	M - H	C16H21NO5	51.5	0.00	3.50	2.83
noroxymorphone	286.1107	2.56	M - H	C16H17NO4	51.2	0.00	0.33	0.07
5-Amino-4-chromanone	274.1108	2.96	M - H, M + FA - H	C15H17NO4	54	0.00	0.08	0.04
Taurohyocholic acid	514.2841	3.17	M - H	C26H45NO7S	40.5	0.00	1.07	0.06
Psychosine sulfate	540.2848	8.65	M - H	C24H47NO10S	41.9	0.00	0.24	0.02
N-Undecylbenzenesulfonic acid	311.1674	9.09	M - H	C17H28O3S	55.6	0.00	1.18	1.86
4-Dodecylbenzenesulfonic Acid	325.1833	10	M - H	C18H30O3S	56.1	0.00	1.24	2.24
LysoPC(16:0)	540.3305	10.09	M + FA - H	C24H50NO7P	56.8	0.00	0.46	0.27
LysoPE(0:0/16:0)	452.2772	10.32	M - H	C21H44NO7P	40.6	0.00	0.24	0.09
LysoPC(18:1)	566.3459	10.46	M + FA - H	C26H52NO7P	58.3	0.00	0.56	0.37
LysoPE(P-16:0)	436.2826	11.01	M - H	C21H44NO6P	53	0.00	0.46	0.28
LysoPC(18:0)	568.3621	11.54	M + FA - H	C26H54NO7P	56.2	0.00	0.47	0.28
LysoPC(O-18:0)	554.3822	12.08	M + FA - H	C26H56NO6P	53.9	0.00	0.62	0.47
DHA	327.2319	12.09	M - H	C22H32O2	41.3	0.00	0.54	0.38
LysoPE(P-18:0)	464.3146	12.31	M - H, 2M - H	C23H48NO6P	41.4	0.00	0.55	0.32
PS(19:1/22:6)	846.5325	14.16	M - H	C47H78NO10P	53.7	0.00	0.49	0.15
PC(20:5/22:6)	896.5469	14.34	M + FA - H	C50H78NO8P	48.1	0.00	0.46	0.18
PC(22:6/18:3)	872.5463	14.42	M + FA - H	C48H78NO8P	48.5	0.00	0.46	0.19
PC(16:1/18:3)	798.5292	14.45	M + FA - H	C42H76NO8P	58.2	0.00	0.64	0.20
PE(19:1/22:6)	848.5476	14.55	M + FA - H	C46H78NO8P	47.8	0.00	0.47	0.15
PE(17:1/22:4)	824.5467	14.61	M + FA - H	C44H78NO8P	57.1	0.00	0.78	0.29
7-Ketodeoxycholic acid	811.5309	14.63	2M - H	C24H38O5	42.3	0.00	0.72	0.39
PE(15:0/22:4)	798.5303	14.68	M + FA - H	C42H76NO8P	49.9	0.00	0.58	0.20
PE(18:0/20:4)	782.5346	14.69	M - H	C43H78NO9P	45.5	0.00	0.40	0.10
PC(15:0/17:2)	774.5305	14.74	M + FA - H	C40H76NO8P	44.6	0.00	0.78	0.37
PC(18:2/22:6)	874.563	14.79	M + FA - H	C48H80NO8P	55.8	0.00	0.49	0.17
PE(20:2/22:6)	814.5347	14.79	M - H	C47H78NO8P	46.9	0.00	0.62	0.34
PE-Cer(22:0/d14:1)	733.5504	14.88	M + FA - H	C38H77N2O6P	54.3	0.00	1.74	2.20
PE(19:0/20:5)	824.5472	14.91	M + FA - H	C44H78NO8P	46.7	0.00	0.90	0.55
PE(16:0/20:2)	788.5402	15.06	M + FA - H	C41H78NO8P	43.6	0.00	0.89	0.63
PE(20:0/20:5)	838.5614	15.13	M + FA - H	C45H80NO8P	45.9	0.00	0.66	0.34
PE(16:0/20:5)	736.4937	15.13	M - H	C41H72NO8P	43.3	0.00	0.66	0.28
PC(18:1/20:5)	850.5626	15.17	M + FA - H	C46H80NO8P	53.8	0.00	0.67	0.33
PE(P-16:0/22:6)	762.5101	15.21	M - H	C43H74NO8P	51.2	0.00	0.70	0.31
PE-Cer(21:0/d16:1)	747.567	15.3	M + FA - H	C39H79N2O6P	50.7	0.00	2.17	3.19
PC(O-16:0/20:5)	810.5667	15.35	M + FA - H	C44H80NO7P	55.5	0.00	0.67	0.30
PS(19:0/22:4)	852.5772	15.37	M - H	C47H84NO10P	57.6	0.00	0.68	0.43
PE(P-16:0/20:5)	720.4974	15.54	M - H	C41H72NO7P	54.4	0.00	1.01	0.61
PC(18:0/20:5)	852.5777	15.64	M + FA - H	C46H82NO8P	50.1	0.00	0.79	0.50
PC(P-18:1/20:4)	836.5818	15.67	M + FA - H	C46H82NO7P	53.5	0.00	0.66	0.34
PC(O-16:0/22:5)	838.5968	15.9	M + FA - H	C46H84NO7P	49.5	0.00	0.59	0.27
GPEtn(18:0/20:5)	764.5247	15.92	M - H	C43H76NO8P	40.5	0.00	0.89	0.51
PI(15:0/22:0)	879.5952	15.95	M - H	C46H89O13P	53.2	0.00	0.68	0.43
PS(21:0/20:4)	898.5784	16.17	M + FA - H	C47H84NO10P	53.1	0.00	1.73	2.69
PC(18:0/22:5)	880.608	16.23	M + FA - H	C48H86NO8P	47.2	0.00	0.61	0.37
PC(O-16:0/22:5)	838.5971	16.23	M + FA - H	C46H84NO7P	45.3	0.00	0.58	0.34
cholestan-oic acid	913.6015	16.37	2M + FA - H	C26H42O5	43.6	0.00	1.40	1.98
PE(21:0/20:3)	856.6073	16.38	M + FA - H	C46H86NO8P	55.7	0.00	0.83	0.62
PE(16:0/18:1)	716.5243	16.41	M - H	C39H76NO8P	57.5	0.00	1.76	1.90
PC(P-18:1/22:5)	862.5921	16.44	M + FA - H	C48H84NO7P	49	0.00	0.55	0.42
PE(P-18:0/20:5)	748.533	16.46	M - H	C43H76NO7P	55.6	0.00	0.97	0.65
Lactosylceramide (16:0/d18:1)	906.6074	16.55	M + FA - H	C46H87NO13	40.8	0.00	0.64	0.44
PE(18:0/20:1)	818.5904	16.57	M + FA - H	C43H84NO8P	40.8	0.00	1.44	1.64
PS(21:0/22:4)	880.6069	16.59	M - H	C49H88NO10P	40.8	0.00	0.70	0.51
PC(O-18:0/22:6)	864.611	16.61	M + FA - H	C48H86NO7P	53.2	0.00	0.44	0.20
DG(18:1/20:5/0:0)	685.5027	16.84	M + FA - H	C41H68O5	47.5	0.02	0.60	0.38
PE(P-18:0/22:6)	774.5461	16.86	M - H	C45H78NO7P	52.3	0.03	0.98	0.66
PE(P-18:0/20:4)	750.5435	17.18	M - H	C43H78NO7P	53.4	0.00	1.60	2.23
PC(P-18:0/20:2)	842.6296	17.29	M + FA - H	C46H88NO7P	52.3	0.00	1.39	1.67
PE(18:1/18:0)	744.5521	17.55	M - H	C41H80NO8P	42	0.00	2.21	3.09
PE(P-16:0/18:0)	702.543	17.59	M - H	C39H78NO7P	47.5	0.00	1.80	2.86
PE(22:0/18:1)	846.6216	17.72	M + FA - H	C45H88NO8P	48.1	0.01	1.62	1.61
PE(O-20:0/19:1)	818.6284	17.95	M + FA - H	C44H88NO7P	51.9	0.00	2.29	2.86

### Analysis of the Significantly Different Metabolites

**FIGURE 8:**
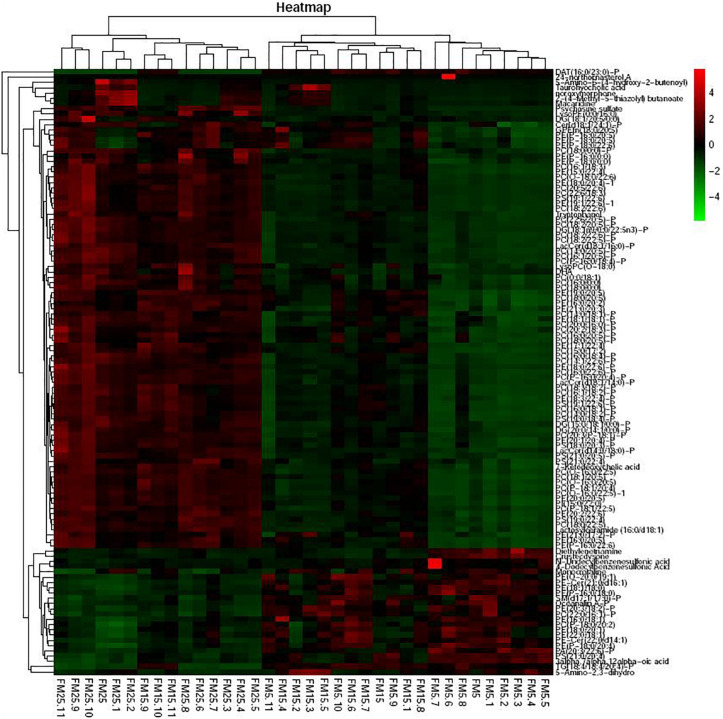
The heatmap of the differential metabolites.

Heatmap based on the significantly different metabolites showed clear difference between shrimp fed the FM25, FM15 and FM5 (Figure 8). Most of these metabolites were lipids, especially phospholipids, including phosphatidylcholine (PC), phosphatidyl ethanolamine (PE), Phosphatidyl serine (PS), Phosphatidyl inositols (PI), phosphatidic acid (PA), sphingolipid, fatty acids, and sterol.

**FIGURE 9:**
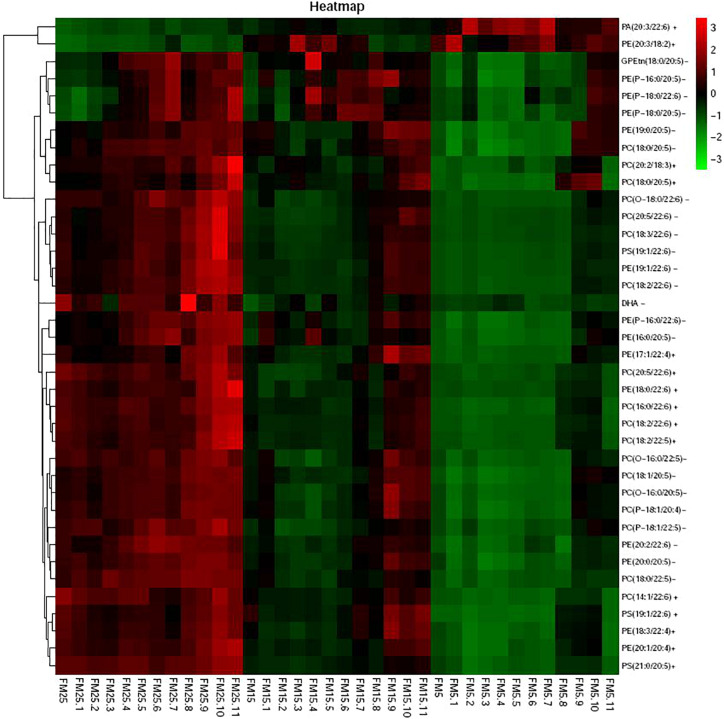
The heatmap of the differential metabolites with unsaturated bond larger than 5.

**FIGURE 10:**
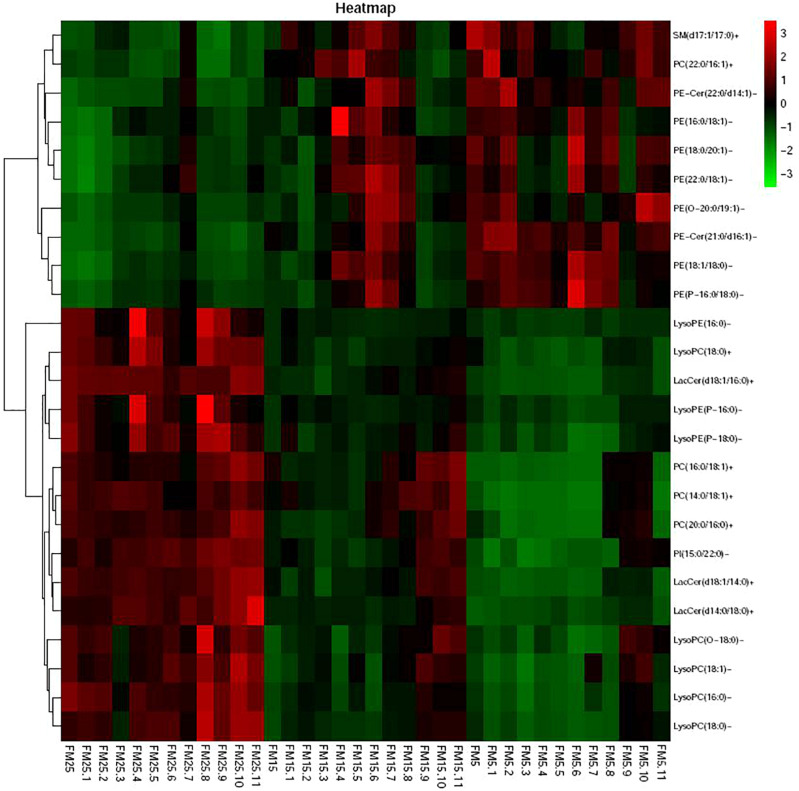
The heatmap of the differential metabolites with 0 or 1 unsaturated bond.

Heatmap based on the lipids with more than five unsaturated bond showed clear differences in three groups (Figure 9), shrimp fed diet with high dietary FM tend to have more polyunsaturated lipids in hemolymph. LysoPCs were higher in hemolymph of shrimp fed high dietary FM (Figure 10), while saturated and monosaturated lipids were tend to be higher in hemolymph of shrimp fed low dietary FM.

**FIGURE 11:**
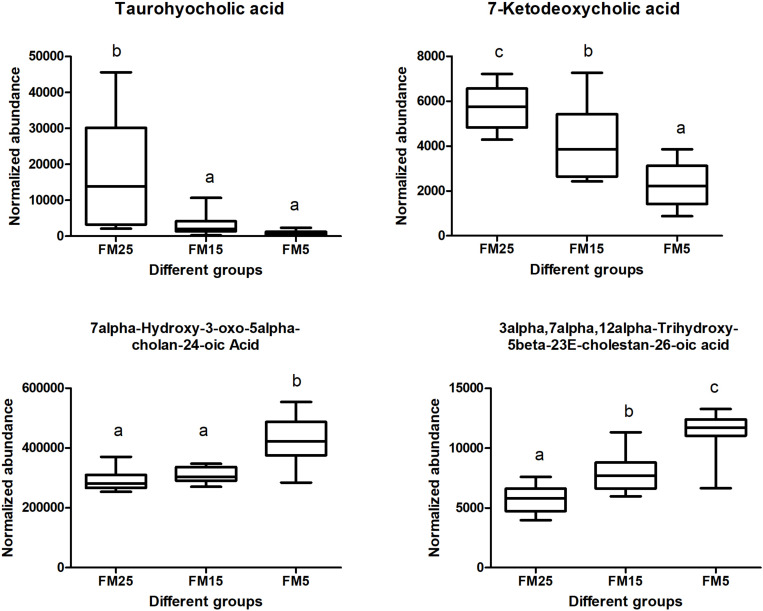
Effect of dietary FM on bile acids contents in hemolymph of white shrimp. Values with different letters denote significantly differences (*P* < 0.05).

Cholesterol and bile acid metabolism were also affected by dietary FM levels in this study (Figure 11). Taurohyocholic acid and 7-Ketodeoxycholic acid were decreased with the decreasing dietary FM levels (*P* < 0.05), while the contents of 7alpha-Hydroxy-3-oxo-5alpha-cholan-24-oic acid and 3alpha,7alpha,12alpha-Trihydroxy-5beta-23E-cholestan-26-oic acid were increased with the decreasing dietary FM levels (*P* < 0.05).

## Discussion

In this study, micro-balanced low FM diets were formulated, phytase and immunoenhancer were supplemented to enhance the digestive and immune ability of white shrimp. Our results revealed that growth performance and feed utilization of *L. vannamei* reared at low salinity was significantly reduced when dietary FM levels were lower than 15%. FM replacement is complex and largely depend on the diet formulation, shrimp quality and the environment. The previous research indicated that minimum FM level in the diet of white shrimp varies widely from 0 to 20%, but most of the research were conducted in sea water, because the nutritional requirement and physiology of white shrimp would be changed at low salinity (Li et al., 2017a), it was hard to compared with our result. Notably, survival of shrimp in this study was decreased with the decreasing FM levels in the diets, which was not observed in most of FM replacement research. The decreased survival in shrimp fed a low FM diet indicated that the low dietary FM harmed the health of shrimp. The impaired intestinal structure and intestinal epithelial cell subcellular structure also proved the above viewpoint. So that the impaired health of shrimp might be the primary problem after dietary FM decreased, but what’s the potential mechanism involved in this process?

Low dietary FM induced ER stress in this study. ER is the primary subcellular organelle of eukaryotic cells, and is the main place for protein folding and maturation, also for lipid synthesis (Tam et al., 2012). Conditions interfering with the function of the ER are collectively called ER stress, which is induced by excessive protein traffic or the accumulation of unfolded protein (Li et al., 2013; Liang et al., 2016). One of the pathway involved in ER stress is double stranded RNA-activated protein kinase like ER kinase (PERK)-eIF2α-ATF4 pathway (Tam et al., 2012). The expression of *eif2*α, *erk* and *atf4* mRNAs in hepatopancreas and intestine were significantly affected by dietary FM levels. Ammonia stress and *WSSV* infection were found to induce the ER stress in white shrimp (Li et al., 2013; Liang et al., 2016; Yuan et al., 2017), while diet induced ER stress of white shrimp was still not reported. ER stress also was observed when rainbow trout was fed plant based diet, the potential reason may be the imbalance of amino acid (Skiba-Cassy et al., 2016). The presence of anti-nutritional factors (gossypol) also induced the ER stress in turbot (Bian et al., 2016). ER stress might be induced by a series of factors, in this study, the amino acids were balanced among all the groups, we were not sure whether the presence of anti-nutritional factors in plant proteins (SPC and wheat gluten) or other factors induced the ER stress.

ER stress was usually coupled with immune response (Tam et al., 2012). NF-κB is a key regulator for immune and inflammatory responses, and the recent research suggested that NF-κB was activated by ER stress during the inflammation (Kitamura, 2011). NF-κB is normally held inactive by its inhibitor, IκBα, and IκBα kinase (IKK) phosphorylate IκBα leading to its degradation and NF-κB activation (Ko et al., 2017). IKK is required for maximum activation of NF-κB in response to ER stress (Tam et al., 2012). In this study, *ikk*β mRNA expression increased in intestine after FM was replaced, which may induced by the ER stress. Dorsal and Relish are the NF-κB family proteins in white shrimp (Qiu et al., 2014), in this study *dorsal* mRNA expression in liver was decreased with the decreasing FM levels, indicated that NF-κB in liver also affected by dietary FM level, and showed the same tendency with ER stress. The development of ER stress and inflammation finally induced intestinal damage in this study, plant proteins were found to have negative effect on the intestinal histology of fish and shrimp in many research, which may caused by the presence of anti-nutritional factors or the imbalance of nutrients (Navarrete et al., 2013; Bansemer et al., 2015). The present study not only observed the impaired intestinal brush border after FM replacement, but also found that reduced the FM in the diet would impaired the subcellular structure of white shrimp (the swollen ER and damaged nucleus).

Low FM impaired the anti-oxidative ability and immune response of white shrimp in our present and previous research (Xie et al., 2016). Although white shrimp is an euryhaline animal, the innate immunity of the white shrimp was weakened by the low salinity farming in many research (Wang and Chen, 2005; Li et al., 2007, 2010). The low salinity farming condition may worsen the low FM stress, and induced lower survival rate in this study. Salinity has a strong influence on energy metabolism, the energy demand of white shrimp would increase at low salinity (Rosas et al., 2002; Ye et al., 2009). In this study, the glucose and lipid metabolism of white shrimp were disturbed by FM replacement. Glucose, TG and TBA in hemolymph were decreased with the decreasing dietary FM, the fatty acid oxidation was decreased and fatty acid synthesis was enhanced in hepatopancreas after dietary FM was decreased. The present results indicated that under low salinity farming condition, shrimp fed the low FM diets may expand more energy on immune response.

Lipids are not only serve as one of the main fuels to supply the energy requirement of animals, also play important role in cell proliferation, immunity, apoptosis and inflammation (Arts and Kohler, 2009). Docosahexaenoic acid (DHA), one of the most important long chain ployunsaturated fatty acids (LC-PUFA), is able to inhibit a number of aspects of inflammation, derivatives of DHA such as resolvins, maresins and protectins also involved in the inflammatory process such as cytokines production and lymphocyte activity (Ye et al., 2010; Zhao et al., 2015). In this study, the DHA in hemolymph decreased with the decreasing dietary FM levels, which indicated shrimp fed the low FM diet consumed more DHA in the immune response. Besides that, lipids also structurally important, as the main component of cellular membranes (Jeromson et al., 2015). Under the stress condition, cell membranes composition and fluidity were associated with the immune response in many studies (Arts and Kohler, 2009). The activity of many membrane-bound proteins were directly regulated by the physical state of membrane lipids, such as ion channels, sensor proteins and receptor-associated protein kinases (Litman et al., 2001; Turner et al., 2003). This fluidity is largely dependent on lipid composition, the addition of fatty acid chain length and acyl chain doubled bonds is generally assumed to increased the membrane fluidity (Stillwell and Wassall, 2003; Maulucci et al., 2016). In this study, the degree of unsaturation of phospholipids in hemolymph of shrimp was decreasing with the decreased dietary FM levels. Most of the phospholipids with five or more double bonds clearly decreased with the decreasing dietary FM levels, especially for the phospholipids incorporated with DHA and EPA. Since LC-PUFA (especially DHA and EPA) are highly flexible, the decreased levels of them in phospholipids would impaired the membrane function, affect the membrane biophysical properties such as fluidity, flexibility and thickness (O’Shea et al., 2009; Kitson et al., 2016). On the other hand, most of phospholipids with 1 or 0 double bonds increased with the decreasing dietary FM levels. The present results clearly indicated the unsaturation degree of fatty acids of phospholipids was decreased in shrimp fed the low FM diet, which means the function of cell membrane in hemolymph was affected by dietary FM levels. The decreased unsaturation degree of fatty acids of phospholipids usually affect the immune receptor in membrane and then induced the impair of following immune response, this might be one of the main reason that health status was affected in shrimp fed a low FM diet. In this study, ER was swollen in shrimp fed a low FM diet, which may also induced by the decreased unsaturation degree of membrane lipids.

lysoPCs (LPC) and lysoPEs (LPE) are derived from PC and PE, they are the most prominent lysoglycerophospholipids and are considered to be inflammatory lipids involved in several immune-mediated disease (Rindlisbacher et al., 2018), and also participate in energy metabolism and storage, cell signaling and other biological processes (Bai et al., 2014). Some research showed that LPC and LPE are increased in inflammation associated diseases, and they exert their effects through different signaling pathways such as PKC, NF-κB and ERK (Zeng et al., 2017). While in other studies, some of the LPC and LPE were decreased in the inflammation condition (Bai et al., 2014; Zhou et al., 2014; Qian et al., 2017; Wang et al., 2017). In this study, several LPC and LPE were decreased with the decreasing dietary FM levels, which indicated the relationship of inflammation and the levels of LPC and LPE may dependent on species and the inflammation stage. In this study, the energy metabolism was affected by dietary FM levels, the disturbed energy metabolism may decreased the LPC and LPE in hemolymph.

Besides the phospholipids metabolism, the bile acid metabolism also affected by dietary FM levels. Bile acids are critical to lipid digestion, cholesterol metabolism and other lipid related pathways (Staley et al., 2017). Disturbance of the bile acid pool may result in a variety of disease states. Taurohyocholic acid is a conjugated primary bile acid (Joyce et al., 2014), in this study, TBA and taurohyocholic acid were decreased with the decreasing dietary FM levels, indicated the lipid digestion was affected, which may related to the impaired lipid metabolism in shrimp fed the low FM diets. 7-Ketodeoxycholic acid is a secondary bile acid and is regarded as toxic bile acid (Chen et al., 2016), while it was decreased with the decreasing dietary FM levels. 7alpha-Hydroxy-3-oxo-5alpha-cholan-24-oic acid and 3alpha,7alpha,12alpha-Trihydroxy-5beta-23E-cholestan-26-oic acid two free primary bile acids, they are the precursor of two cytotoxicity bile acids deoxycholic acid and lithocholic acid (Chen et al., 2016), their increase in hemplymph may associated with the inflammation in shrimp fed low FM diets. These results clearly indicated the bile acids metabolism was affected by dietary FM levels, which may closely linked to the impaired lipid metabolism and inflammation in shrimp fed a low FM diets.

## Conclusion

In conclusion, at the low salinity farming condition, low dietary FM would decrease the growth performance and feed utilization of shrimp, also affect the health status of shrimp. The poor growth performance and unhealthy status of shrimp fed a low FM diet may mainly caused by the decreased unsaturated degree of membrane phospholipids, decreased contents of lysophospholipid and the disturbed lipid metabolism.

## Data Availability Statement

The raw data supporting the conclusions of this article will be made available by the authors, without undue reservation, to any qualified researcher.

## Author Contributions

SX and LT designed the experiments. SX carried out the experiments and wrote the manuscript. JN, YL, and BT reviewed and revised the manuscript. All authors contributed to the article and approved the submitted version.

## Conflict of Interest

The authors declare that the research was conducted in the absence of any commercial or financial relationships that could be construed as a potential conflict of interest.
