# Supplementary material for: Low Dietary Fish Meal Induced Endoplasmic Reticulum Stress and Impaired Phospholipids Metabolism in Juvenile Pacific White Shrimp, Litopenaeus vannamei
Source: Front Physiol. 2020 Aug 18;11:1024. doi: 10.3389/fphys.2020.01024 (PMC7462021; doi:10.3389/fphys.2020.01024)
Supplement: Supplementary file 1 [file Table_1.docx]

**Supplementary Table 1** The amino acids composition (%) of the ingredients.

|  | Fish meal | Soyben meal | Peanut meal | SPC | Wheat meal | Wheat gluten | Blood meal | Chicken meal |
| --- | --- | --- | --- | --- | --- | --- | --- | --- |
| Taurine | 0.628 | 0.029 | 0.023 | 0.013 | 0.083 | 0.011 | 0.05 | 0.211 |
| Asp | 6.074 | 5.055 | 5.747 | 6.965 | 0.516 | 2.603 | 9.792 | 5.678 |
| Thr | 2.788 | 1.761 | 1.376 | 2.43 | 0.326 | 1.927 | 2.653 | 2.196 |
| Ser | 2.555 | 2.309 | 2.423 | 3.084 | 0.577 | 3.578 | 3.78 | 2.345 |
| Glu | 9.49 | 8.433 | 9.652 | 11.97 | 4.004 | 24.274 | 10.201 | 8.954 |
| Gly | 4.206 | 1.991 | 2.733 | 2.673 | 0.455 | 2.553 | 4.498 | 4.822 |
| Ala | 4.159 | 2.01 | 2.042 | 2.715 | 0.372 | 2.053 | 6.421 | 3.481 |
| Cys | 0.686 | 0.664 | 0.739 | 0.796 | 0.263 | 1.271 | 0.694 | 0.611 |
| Val | 3.522 | 2.257 | 2.399 | 3.353 | 0.564 | 3.144 | 6.44 | 2.91 |
| Met | 1.819 | 0.482 | 0.572 | 0.735 | 0.027 | 0.824 | 0.506 | 1.181 |
| Ile | 2.986 | 2.104 | 1.912 | 3.076 | 0.418 | 2.782 | 0.31 | 2.445 |
| Leu | 4.95 | 3.507 | 3.461 | 4.912 | 0.838 | 5.267 | 10.291 | 4.159 |
| Tyr | 2.065 | 1.727 | 2.034 | 2.386 | 0.352 | 2.711 | 1.933 | 1.754 |
| Phe | 2.821 | 2.343 | 2.667 | 3.313 | 0.596 | 3.993 | 6.049 | 2.447 |
| His | 1.77 | 1.231 | 1.245 | 1.698 | 0.285 | 1.73 | 5.216 | 1.435 |
| Lys | 4.988 | 2.862 | 2.001 | 4.031 | 0.266 | 1.327 | 7.312 | 3.548 |
| Arg | 3.876 | 3.243 | 5.447 | 4.439 | 0.426 | 2.575 | 3.594 | 3.922 |
| Pro | 3.205 | 2.446 | 2.393 | 3.355 | 1.378 | 9.51 | 3.237 | 3.785 |
| Total | 62.586 | 44.455 | 48.866 | 61.95 | 11.746 | 72.131 | 82.977 | 55.882 |

**Supplementary Table 2** The fatty acid composition of different diets (% total fatty acids).

|  | Diets |  |  |  |  |
| --- | --- | --- | --- | --- | --- |
| Fatty acid | 1 | 2 | 3 | 4 | 5 |
| C12:0 | 0 | 0.057 | 0.076 | 0.08 | 0.082 |
| C14:0 | 2.6 | 2.7 | 2.8 | 2.5 | 2.5 |
| C15:0 | 0.14 | 0.15 | 0.15 | 0.18 | 0.17 |
| C16:0 | 15.2 | 16 | 16.1 | 16.3 | 16.7 |
| C17:0 | 0.18 | 0.2 | 0.26 | 0.27 | 0.27 |
| C18:0 | 4.1 | 4.4 | 4.7 | 4.6 | 4.6 |
| C24:0 | 0.25 | 0.23 | 0.4 | 0.45 | 0.45 |
| C16:1 | 4.2 | 4 | 4.1 | 4.1 | 3.9 |
| C17:1 | 0.5 | 0.45 | 0.36 | 0.35 | 0.34 |
| C18:1 | 24.6 | 25.1 | 25.5 | 26 | 26.2 |
| C20:1 | 1.3 | 1.3 | 1.3 | 1.5 | 1.4 |
| C22:1 | 0.15 | 0.17 | 0.21 | 0.19 | 0.17 |
| C24:1 | 0.28 | 0.3 | 0.26 | 0.22 | 0.21 |
| C18:2 | 28.8 | 28.6 | 28 | 27.6 | 27.4 |
| C20:2 | 0.29 | 0.37 | 0.28 | 0.27 | 0.3 |
| C22:2 | 0.28 | 0.25 | 0.24 | 0.25 | 0.23 |
| C18:3 | 4.5 | 4.1 | 4.1 | 4.2 | 4.1 |
| C20:3 | 0.66 | 0.7 | 0.72 | 0.7 | 0.72 |
| C18:4 | 0.72 | 0.65 | 0.58 | 0.57 | 0.56 |
| C20:4 | 0.75 | 0.71 | 0.62 | 0.69 | 0.63 |
| C22:4 | 0.29 | 0.25 | 0.22 | 0.22 | 0.2 |
| C20:5 | 5.8 | 5.4 | 5.2 | 5.1 | 5.1 |
| C22:5 | 1.5 | 1.3 | 1.4 | 1.3 | 1.3 |
| C22:6 | 2.4 | 2.3 | 2.2 | 2.1 | 2.1 |
| SFA | 23.47 | 25.737 | 27.486 | 28.38 | 29.772 |
| MUFA | 31.03 | 31.32 | 31.73 | 32.36 | 32.22 |
| PUFA | 45.99 | 44.63 | 43.56 | 43 | 42.64 |

SFA, saturated fatty acid, C12:0, C14:0, C15:0, C16:0, C17:0, C18:0, C24:0;

MUFA, mono-unsaturated fatty acid, C16:1, C17:1, C18:1, C20:1, C22:1, C24:1;

PUFA, poly-unsaturated fatty acid, C18:2, C20:2, C22:2, C18:3, C20:3, C18:4, C20:4, C20:5, C22:5, C22:6;

**Supplementary Table 3** Real-time PCR primer sequences

| Target genes | Primer sequence (5' to 3') |
| --- | --- |
| Actin F | GAGCAACACGGAGTTCGTTGT |
| Actin R | CATCACCAACTGGGACGACATGGA |
| EF1αF | TGGCTGTGAACAAGATGGAC |
| EF1αR | AGATGGGGATGATTGGGACC |
| eIF2αF | GAATAAACCTAATCGCACCACC |
| eIF2αR | CTAATGCCCTAAGACCATCCTG |
| ERK F | GCTGAACTCAAAGGGC |
| ERK R | GGGAACAGTGGACGGT |
| ATF4 F | GAAGTCTGGAGCTGGAGCATCA |
| ATF4 R | CAGGGACTCCAAAGGATGCTT |
| Dorsal F | TGGGGAAGGAAGGATGC |
| Dorsal R | CGTAACTTGAGGGCATCTTC |
| IKKβ F | TGTGGTTTACGAGAGGCT |
| IKKβ R | GTTCCAACAAAGGAGGTG |
| Relish F | CTACATTCTGCCCTTGACTCTGG |
| Relish R | GGCTGGCAAGTCGTTCTCG |
| CPT-1 F | CAACTTCTACGGCACTGAT |
| CPT-1 R | GTCGGTCCACCAATCTTC |
| Glut-2 F | TCTAGTGAGTCTCGGAGTC |
| Glut-2 R | GGTCCGTAGCCGATACTG |
| FAS F | CAGGTGGAGATGCTCCTCGTGTT |
| FAS R | GGTGACTAGCTCGGCTACATGGTT |
| AMPK F | TCAGAGGAGGAGCAGGAAC |
| AMPK R | CCCGAGGTCTAATAGGCAC |
| AK F | ACTCCTCACCCTGGCTCCT |
| AK R | GGTTTGTCATCTTCCCTTTG |
| PK F | CTCACTTTCTGCCCCACC |
| PK R | TGTACTTGCCAGCGACCT |

EF1α, elongation factor 1α; eIF2α, eukaryotic initiation factor 2 alpha; ATF4, activating transcription factor 4; ERK, extracellular signal-regulated kinase; IKKβ, inhibitor of nuclear factor kappa-B kinase β; CPT-1, carnitine palmitoyltransferase 1; FAS, fatty acid synthetase; AMPK, denosine monophosphate activated protein kinase; AK, arginine kinase; PK, pyrubate kinase; Glut2, glucose transporter 2.
